# Compulsivity is measurable across distinct psychiatric symptom domains and is associated with familial risk and reward-related attentional capture

**DOI:** 10.1017/S1092852919001330

**Published:** 2020-08

**Authors:** Lucy Albertella, Samuel R. Chamberlain, Mike E. Le Pelley, Lisa-Marie Greenwood, Rico SC Lee, Lauren Den Ouden, Rebecca A. Segrave, Jon E. Grant, Murat Yücel

**Affiliations:** 1 School of Psychological Sciences and Turner Institute for Brain and Mental Health, Monash University, Clayton, Victoria, Australia; 2 Department of Psychiatry, University of Cambridge, Cambridge, United Kingdom; 3 Department of Psychiatry, Cambridge and Peterborough NHS Foundation Trust, Cambridge, United Kingdom; 4 School of Psychology, UNSW Sydney, New South Wales, Australia; 5 School of Psychology, University of Wollongong, Wollongong, New South Wales, Australia; 6 Illawarra Health and Medical Research Institute, University of Wollongong, Wollongong, New South Wales, Australia; 7 Department of Psychiatry and Behavioral Neuroscience, University of Chicago, Chicago, Illinois, USA

**Keywords:** Addiction, compulsive, phenotype, marker, cognition.

## Abstract

**Background.:**

Compulsivity can be seen across various mental health conditions and refers to a tendency toward repetitive habitual acts that are persistent and functionally impairing. Compulsivity involves dysfunctional reward-related circuitry and is thought to be significantly heritable. Despite this, its measurement from a transdiagnostic perspective has received only scant research attention. Here we examine both the psychometric properties of a recently developed compulsivity scale, as well as its relationship with compulsive symptoms, familial risk, and reward-related attentional capture.

**Methods.:**

Two-hundred and sixty individuals participated in the study (mean age = 36.0 [SD = 10.8] years; 60.0% male) and completed the Cambridge-Chicago Compulsivity Trait Scale (CHI-T), along with measures of psychiatric symptoms and family history thereof. Participants also completed a task designed to measure reward-related attentional capture (*n* = 177).

**Results.:**

CHI-T total scores had a normal distribution and acceptable Cronbach’s alpha (0.84). CHI-T total scores correlated significantly and positively (all *p* < 0.05, Bonferroni corrected) with Problematic Usage of the Internet, disordered gambling, obsessive-compulsive symptoms, alcohol misuse, and disordered eating. The scale was correlated significantly with history of addiction and obsessive-compulsive related disorders in first-degree relatives of participants and greater reward-related attentional capture.

**Conclusions.:**

These findings suggest that the CHI-T is suitable for use in online studies and constitutes a transdiagnostic marker for a range of compulsive symptoms, their familial loading, and related cognitive markers. Future work should more extensively investigate the scale in normative and clinical cohorts, and the role of value-modulated attentional capture across compulsive disorders.

## Introduction

Compulsivity refers to the tendency towards undertaking repetitive, habitual actions, whereby the original goal of the act has been lost^1^ (cf. Reference 2). For example, an individual with obsessive-compulsive disorder (OCD) may repeatedly check that the gas stove has been switched off for hours per time, despite only recently having already checked that it was indeed switched off. While OCD is the classic archetypal disorder of compulsivity, it is increasingly recognized that mental disorders listed in non-OCD DSM diagnostic categories also have compulsive features, notably gambling disorder, substance addictions, and binge-eating disorder.^3^ ^–^^5^ These conditions collectively share a number of parallels including high rates of comorbid expression. In order to better understand the common etiological and biological predisposing factors towards these compulsive symptom types, it is necessary to identify transdiagnostic markers that cut across conventionally separate conditions. By identifying latent phenotypes that are dimensional in nature, existing in milder forms in the background population, and in more extreme forms across mental disorders, it is hoped that progress can be made in improving early detection, diagnostic classification systems, neurobiological models, and treatment approaches.^6^ ^–^^8^

The Cambridge–Chicago Compulsivity Trait Scale (CHI-T) is a 15-item scale that was recently developed to measure a broad range of compulsive traits. In an initial validation study conducted using in-person clinical assessments, the CHI-T had good psychometric properties, with total scores occupying a normative distribution, and convergent validity being demonstrated against relevant symptoms (correlating with OCD, gambling disorder, and substance use disorder symptoms).^5^

To demonstrate the utility of the CHI-T scale as a transdiagnostic measure of compulsivity, it is important to show that it is associated with familial risk of manifest compulsive disorders, ideally also showing it is not associated with noncompulsive disorder(s). Research has consistently shown family history of addiction to be associated with addictive behaviors^9^ ^–^^11^ and family history of OCD to be associated with OCD diagnosis and/or symptoms.^12^ ^,^^13^ However, no study to date has examined the relationship between familial risk and compulsivity transdiagnostically. This is likely related to the historical lack of a compulsivity measure that can be applied across different behavioral domains and that is sensitive to individual variations in compulsivity along a continuum in the general population.

High levels of compulsivity traits would also be expected to share some degree of neurocognitive correlates with addictive and compulsive disorders. Specifically, these neurocognitive markers may reflect processes that put individuals at risk of, or provide a predisposition toward, developing a range of compulsive behaviors. One cognitive risk marker that has been linked in various ways to compulsive addiction-related behaviors is the tendency to show strong attentional biases and approach responses toward reward-related cues, also known as “sign-tracking”.^14^ Sign-tracking (tracking the signal), in contrast to goal-tracking, that is, approaching the location of reward delivery (tracking the goal), is thought to reflect an individual’s propensity to attribute incentive salience to Pavlovian signals of reward, such that these reward-signalling cues become attractive in their own right and can powerfully influence subsequent behavior and viewed as reflecting propensity to develop addictive behaviors.^15^ ^,^^16^ Importantly, while sign-tracking is generally recognized as a conditioned behavior directed toward reward cues, reward cues are not the only stimuli capable of eliciting a sign-tracking response. Safety signals, that is, stimuli that signal the omission of an expected aversive event (such as shock), also elicit a sign-tracking response, ^17^ suggesting that they also may be endowed with incentive salience (through their relationship with the absence of threat) and thereby capable of attracting attention and approach responses in their own right. For example, in contamination-based OCD, washing-related stimuli (eg, soap) may become safety signals through their pairing with reduced contamination threat and anxiety. In turn, these stimuli can acquire incentive salience (for sign-trackers), drawing attention and approach in their own right. To the extent that certain individuals attribute incentive salience to Pavlovian cues (reward-related or safety-related), such that associated behaviors may continue independently of the outcome, these individuals may be argued to be at risk of developing maladaptive behaviors. From this perspective, sign-tracking may be viewed as a transdiagnostic risk marker for compulsive or otherwise maladaptive cue-driven behaviors (for a more detailed account of this model, see^18^).

**Figure 1.**
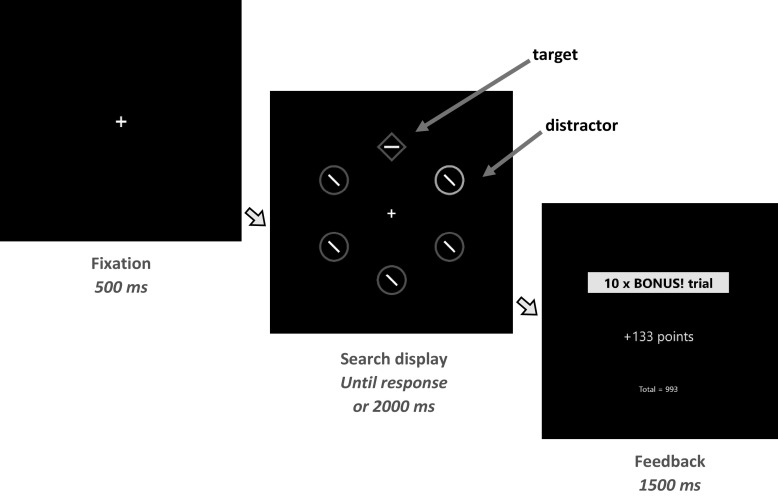
Sequence of trial events in the visual search task. Participants responded to the orientation of the line segment (horizontal or vertical) within the diamond (target). One of the nontarget circles could be a colour singleton distractor. Fast, correct responses to the target received monetary reward, depending on the distractor colour. A distractor rendered in a high-reward colour signalled that this was a bonus trial on which a large reward could be won. If instead the search display contained a distractor rendered in a low-reward colour (or did not contain a colour singleton distractor), then the trial was a standard trial on which only a small reward was available. Slower response times (RTs) on trials with a high-reward distractor than trials with a low-reward distractor demonstrate value-modulated attentional capture (VMAC).

While much of the research on sign-tracking has used animal models, Le Pelley et al.^19^ developed a procedure to assess an analogue of sign-tracking in human attention. This involved a visual search task, in which participants searched for and responded to a diamond target among circles on every trial (see Figure 1). Critically, one of the (nontarget) circles could be colored, either blue or orange (all other shapes were grey). The color of this color-singleton circle—referred to as the *distractor*—related directly to the size of the reward available on the current trial: one color (the high-reward color) signaled that a large reward was available for a correct response, and the other (low-reward) color signaled that a small reward was available. Notably, while the distractor signaled reward magnitude, it was not the target that participants responded to in order to receive that reward; thus distractors had a Pavlovian, but not instrumental, relationship with reward. The key finding was that responses to the target were significantly slower (but no more accurate) for trials with a high-reward distractor compared to trials with a low-reward distractor. This suggests that the signal of high reward was more likely to capture participants’ attention, slowing their response to the target—even though this enhanced capture was counterproductive, because it meant participants earned less on high-reward trials than would otherwise have been the case. This effect of reward on distraction is referred to as *value-modulated attentional capture* (VMAC) and may be considered to reflect the extent to which reward-signals come to influence behavior; that is, the propensity towards “attentional sign-tracking”. Consistent with the idea that this task may be considered an analogue of sign-tracking in animals, individual differences in VMAC have been linked to addictive behaviors.^20^

Thus, the aims of the current study were to: (i) further examine the CHI-T scale, and its relationship with relevant compulsive symptoms; (ii) examine CHI-T’s sensitivity to familial risk of compulsive symptoms; and (iii) understand CHI-T’s relationship with reward-related attentional capture, a cognitive process theorized to be crucial in compulsivity.

## Material and Methods

### Participants

Adult participants, aged 18–80 years, were recruited via Mechanical Turk, for a study advertised as exploring compulsivity. Mechanical Turk is a commonly used online recruitment tool for collecting data, in which individuals complete tasks for a set fee. It offers potential advantages over other recruitment methods in terms of rapidity of data collection; furthermore, Mechanical Turk workers are demographically diverse.^21^ ^,^^22^

Prior to taking part, each individual provided consent after reading the study information sheets, and proceeded to complete the online survey. Following completion of the survey, each participant received payment of $9 (US) ($6 plus bonus of $3). Exclusion criteria were: not willing to consent, out of age range, or not completing the survey. All study procedures were carried out in accordance with the Declaration of Helsinki. The Monash University Human Research Ethics Committee ethically reviewed and approved the study.

### Online measures

All measures and tasks were presented using Inquisit. The following demographic information was collected: age, country of birth, gender, and ethnicity. Additionally, the following questionnaires were administered:

• The Cambridge-Chicago Compulsivity Trait Scale (CHI-T).^5^ This is a 15-item scale covering broad aspects of compulsivity including the need for completion or perfection, reward-seeking, desire for high standards, and avoidance of situations that are hard to control. For each item, participants selected whether the statement applied to them by selecting “strongly disagree,” “disagree,” “agree,” or “strongly agree,” scored as 0–3 respectively. The measure of interest was the total score.
• Obsessive-Compulsive Inventory Revised (OCI-R).^23^ This is a previously validated 18-item scale enquiring about OC-related experiences over the preceding month. For each item the individual rated how distressed or bothered they had been by this over the past month (not at all, a little, moderately, a lot, or extremely, scored 0–4 respectively). The measure of interest was the total score.
• Young’s Internet Addiction Test (IAT) Short Version.^24^ This 12-item questionnaire was developed to measure problematic usage of the Internet. For each of 12-items (eg, ‘How often do you find that you stay online more often than intended?) the participants were asked to rate this over the past month. For each item, the response options were: never, rarely, sometimes, often, or very often, scored 0–4 respectively. The measure of interest was the total score.
• Problem Gambling Severity Index (PGSI) is a 9-item measure of problem gambling severity (derived from the 31-item Canadian Problem Gambling Index^25^). For each item, the response option was: never, sometimes, most of the time, or always, scored 0–3 respectively. Total score was the measure of interest.
• Brief Assessment Tool for Compulsivity Associated Problems (BATCAP).^18^ This is a recently developed tool designed to quantify relevant features of a range of compulsive symptom types, within the auspices of a single convenient instrument. Symptom domains were: alcohol use, gambling, compulsive eating, contamination compulsions, checking compulsions, just right and ordering compulsions, and compulsive Internet use. For each of these types of symptoms, individuals answered 6 questions^1^ covering time lost, distress, loss of control, functional impact, anxiety if prevented from doing the behavior, and strongest urge. Items 1–5 were adapted from the Yale-Brown Obsessive Compulsive Scale^26^ and Florida Obsessive-Compulsive Inventory.^27^ Item 6 was adapted from the Penn Alcohol Craving Scale.^28^ Each was rated on a 5-point scale, scored 0–4 respectively. The measure of interest here was total score for each symptom domain, and participants who had not endorsed a behavior in the past month were given 0 for that domain.
• Family History of Compulsive Behaviors Scale. This is a 12-item scale designed to measure the presence of a range of compulsive behaviors and conditions, past or current, in first-degree family members. Response options range from 0 (no relatives) to 2 (multiple first-degree relatives) for each item. Six items ask about addiction-related behaviors/conditions (alcohol, gambling, and binge eating) and 7 items ask about OCD and related conditions (OCD subtypes, hoarding, obsessive compulsive personality disorder, body-focused repetitive behaviors, and tics). The measures of interest were the 2 subscale (addictions vs. OCD-related) total scores. As a control measure, we also asked about family history of psychosis and schizophrenia (yes/no). The Family History of Compulsive Behaviors Scale is presented in the supplementary materials.
• Psychological Distress. Participants completed the brief Depression Anxiety Stress Scales (DASS-21).^29^ The DASS-21 contains 21 items assessing depression, anxiety, and stress/tension symptoms over the past week. The measure of interest was total score, reflecting general psychological distress.
• *Short UPPS-P Impulsivity Scale* (S-UPPS-P).^30^ This is a 20-item scale that measures impulsivity with 5 subscales: Negative Urgency, the tendency toward impulsive action when experiencing strong negative emotions; Positive Urgency, the tendency toward impulsive action when experiencing strong positive emotions; Lack of Perseverance; Lack of Premeditation; and Sensation Seeking. The current study used the total score, a measure of trait impulsivity, to control for its possible confounding influence on the relationship between CHI-T score and value-modulated attentional capture.

### Value-modulated attentional capture task – reward-only variant

The visual search task used a reward-only variant of Le Pelley et al.’s^19^ (Experiment 2) VMAC procedure, modified to reflect reward-related attentional capture more specifically^2^.

All stimuli were presented on a black background. Each trial began with a central fixation cross, followed after 500 ms by the search display. The search display comprised 6 shapes—5 circles, and one diamond (the target)—arranged evenly around an imaginary ring (see Figure 1). Color set was blue and orange, with assignment of blue and orange to the roles of high-reward and low-reward colors being counterbalanced across participants. The diamond target contained a white line segment oriented either vertically or horizontally; all other shapes contained the same line segment tilted 45° randomly to the left or right. Participants’ task was to report the orientation of the line within the target as quickly as possible—by pressing either the ‘C’ key (horizontal) or ‘M’ key (vertical)—with faster responses earning more points.

Each trial-block of the task comprised 25 trials: 11 trials featuring a distractor rendered in the high-reward color, 11 trials with a distractor in the low-reward color, and 3 distractor-absent trials (in which all shapes were grey), in random order. For correct responses, on trials with a low-reward distractor and distractor-absent trials, participants won 0.1 points for every ms that their response time (RT) was below 1000 ms (so an RT of 600 ms would earn 40 points). Trials in which the display contained a high-reward distractor were labeled as bonus trials, and points were multiplied by 10 (so an RT of 600 ms would earn 400 points). Correct responses with RT greater than 1000 ms and incorrect responses earned no points. The search display remained on-screen until the participant responded or the trial timed-out (after 2 s). A feedback screen then appeared. On ‘standard’ (low-reward distractor or distractor-absent) trials, if the response was correct, feedback showed the number of points earned on that trial; if the response was incorrect, feedback showed “ERROR”; and if the trial timed-out feedback was “TOO SLOW: Please try to respond faster”. On bonus (high-reward) trials the corresponding feedback was accompanied by a box labeled “10 × bonus trial!.” Target location, distractor location, and target line segment orientation (vertical or horizontal) were randomly determined on each trial.

Participants were informed that the aim of the visual search task was to earn as many points as possible, and that they could receive a bonus $3 based upon their performance. Participants were further informed (1) that when a circle in the high-reward color was present in the search display it would be a bonus trial on which points were multiplied by 10, and (2) that when a circle in the low-reward color was present it would not be bonus trial. Participants completed five 25-trial blocks, taking a break between blocks; during this break they were shown the total number of points they had earned so far.

Typically, overall accuracy in this type of visual search task is relatively high, and analyses focus on differences in response time.^19^ ^,^^31^ ^,^^32^ Following this approach, to assess the effect of reward, we calculated a *VMAC score* for each participant by subtracting response time on trials with a low-value distractor from response time on trials with a high-value distractor. A higher VMAC score indicates greater distraction by the high-reward distractor relative to the low-reward distractor; that is, a greater influence of reward on attentional capture. Only correct responses were analyzed, and participants with less than 50% overall accuracy were excluded. Since we were interested in the effect of reward on steady-state behavior, we calculated VMAC scores using data from the final 2 blocks (50 trials in total), when participants had had considerable experience of the color–reward relationships—as in previous research using the VMAC task.^33^

### Data analysis

Distributions of CHI-T total scores were characterized graphically in terms of any skew and outliers. Psychometric properties of the CHI-T were examined (Cronbach’s alpha). Simple relationships between CHI-T total scores and the other measures of interest were explored using correlation analyses (Spearman’s *r*). We report correlation *p*-values uncorrected, two-tailed, but these were only deemed statistically significant if they withstood Bonferroni correction for the number of measures examined per category of interest. In order to identify measures associated with CHI-T scores across the range of manifest compulsive symptom domains, controlling for inter-relationships across such measures (including general distress), secondary analysis was conducted using the statistical technique of partial least squares (PLS).^34^ ^,^^35^ PLS is a versatile multivariate technique that optimally explains relationships between a set of explanatory (X) and output (Y) variables. PLS offers advantages over conventional statistical approaches in that it is robust even when normal assumptions are violated (eg, in situations of collinearity); and is suitable even when there are a relatively large number of variables in comparison to the sample size. The PLS model was fitted using leave-one-out cross-validation (non-linear iterative partial least squares, NIPALS algorithm), and the optimal number of latent factors was selected by minimizing the predictive residual sum of the squares (PRESS). X variables significantly contributing to the model (ie, explaining significant variance in CHI-T scores, Y variable) were identified on the basis of 95% confidence intervals for bootstrap distribution of the standardized model coefficients not crossing zero (*N* = 2500 bootstraps). All analyses were conducted using JMP Pro software version 13.2.

The relationship between CHI-T and value-modulated attentional capture was assessed using correlation analyses, including partial correlation analysis controlling for psychological distress (DASS-21) and impulsivity (S-UPPS-P). Psychological distress and impulsivity were controlled for due to past research showing that these variables can influence compulsive behaviors^36^ ^–^^39^ as well as reward-related learning, ^31^ ^,^^40^ ^,^^41^ and thereby have confounding potential.

## Results

**Figure 2.**
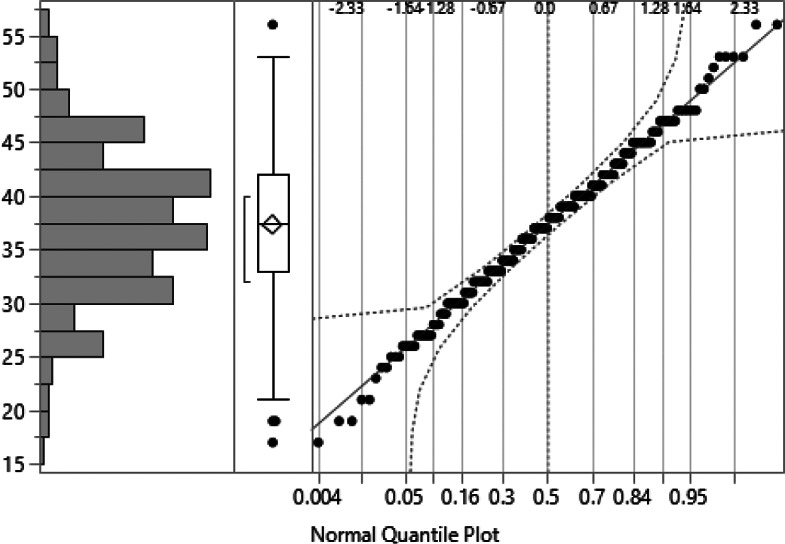
Distribution of CHI-T total scores in the sample. Left—histogram; middle—box-whisker plot (the red bracket defines the shortest half of the data ie, the densest region); and right—Normal Quantile Plot.

The overall sample comprised 260 individuals, of mean (standard deviation) age 36.0 (10.8) years, being 60.0% male, the overwhelming majority (>95%) of USA origin. The CHI-T total scores yielded a normal distribution with few outliers (*n* = 4 out of 260); see Figure 2. Cronbach’s alpha was 0.84, with all individual scale items exhibiting strong loading onto all other items (all alpha >0.82). Of the 260 participants, 44 participants did not proceed to the VMAC task. Of those who did, 10 participants did not finish it, and 29 achieved less than 50% accuracy (ie, numerically below chance). The remaining sample (*N* = 177) was used in the analyses involving VMAC scores.

CHI-T total scores were not correlated with age (*r* = –0.0610, *p* = 0.3271), nor did they differ as a function of gender (*F* [*df* = 1,258] = 0.8708, *p* = 0.3516), or ethnic group (*F* [*df* = 10,249] = 0.8588, *p* = 0.5725). Correlations between CHI-T total scores and different compulsive symptom types are summarized in Table 1, where it can be seen that CHI-T scores correlated significantly and positively (all *p* < 0.05 with Bonferroni correction) with Problematic Usage of the Internet, disordered gambling, obsessive-compulsive symptoms, alcohol misuse, and disordered eating. CHI-T scores also correlated with psychological distress (DASS-21) as expected (*r* = 0.4495, *p* < 0.001). Table 1 also shows the correlations between CHI-T total score and family history of addiction and OCD-related disorders. A higher CHI-T score was significantly associated both with a greater family history score of addictions and a greater family history score of OCD-related disorders (both *p* < 0.05). CHI-T scores did not differ significantly between those participants with and without a history of psychosis or schizophrenia in a first-degree relative (*F* [*df* = 16,225] = 2.5721, *p* = 0.1101).

**Table 1.** Correlations between CHI-T total scores and different compulsive symptom domains.

Measure	*r*	*p*
**Conventional scales**		
IAT total score	0.3599	<.0001
PGSI total score	0.1777	0.0041
OCI-R total score	0.5234	<0.001
**BATCAP **		
Compulsive Alcohol Use	0.1627	0.0086
Compulsive Gambling	0.1980	0.0013
Compulsive Eating	0.1793	0.0037
Contamination compulsions	0.2820	<.0001
Checking compulsions	0.3644	<.0001
Just right and ordering compulsions	0.3846	<.0001
Problematic Usage of the Internet	0.3519	<.0001
**Family history (first-degree relatives)**		
Family history of addictions	0.200	0.0030
Family history of OCRDs	0.2487	<.001

Abbreviations: BATCAP, Brief Assessment Tool for Compulsivity Associated Problems; IAT, Internet addiction test; PGSI, Pathological Gambling Symptoms Inventory; OCI-R, Obsessive Compulsive Inventory Revised; OCRDs, obsessive-compulsive and related disorders.

*p*-values are uncorrected.

**Figure 3.**
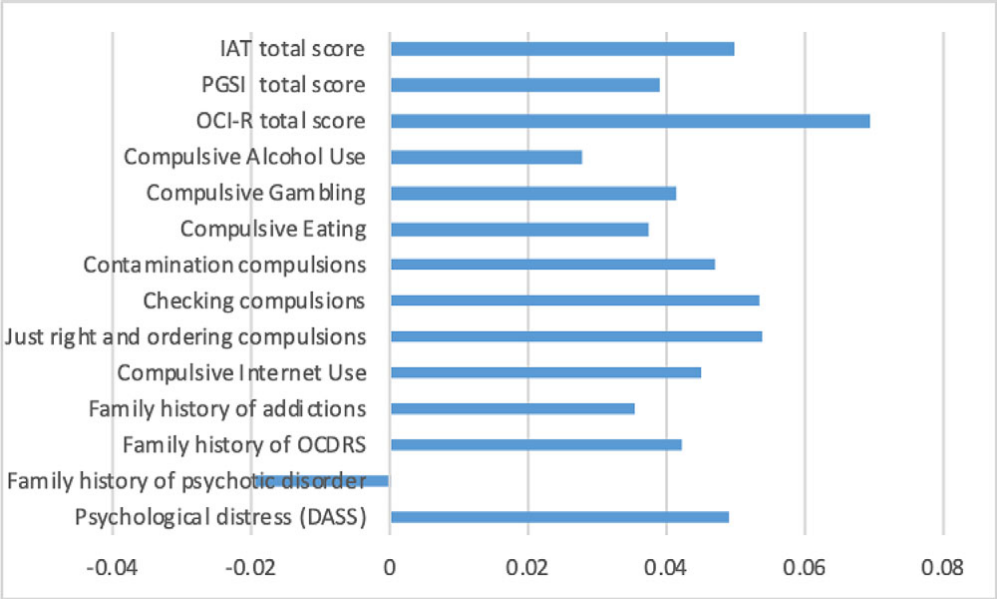
Standardized model coefficients for PLS model, linking each explanatory (X) variable to CHI-T scores (Y). All explanatory variables were statistically significant predictors of higher CHI-T scores (*p* < 0.05, bootstrap) except for family history of psychotic spectrum disorder.

PLS identified an optimal model with one latent factor, accounting for 44.7% of variance in the explanatory (X) measures (ie, compulsive symptom scores, family history of addiction or psychosis, and psychological distress) and 16.8% of variation in CHI-T total scores. Higher levels of each type of compulsive symptom were statistically significant predictors of higher CHI-T scores, as was family history of addiction and OC-related disorders, and general distress (Figure 3; each *p* < 0.05 by bootstrap). Family history of psychosis/schizophrenia was not a statistically significant predictor of CHI-T scores.

**Figure 4.**
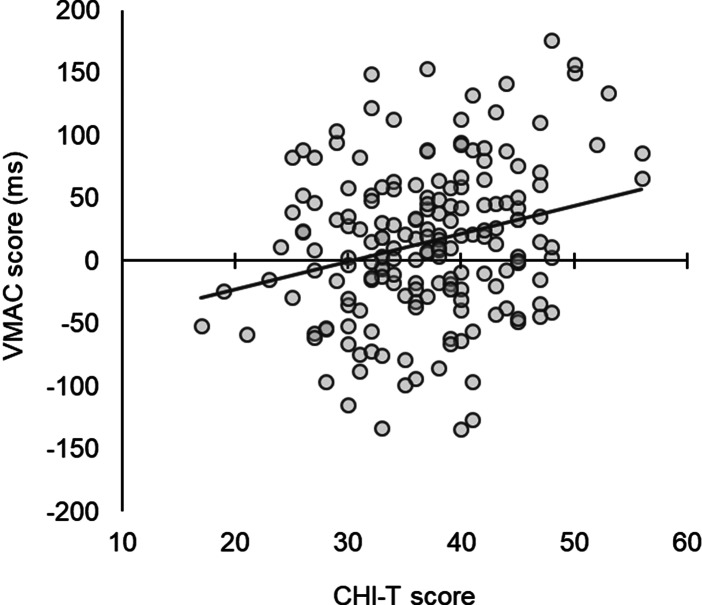
A scatterplot of VMAC score (response time for trials with a distractor that signaled high-reward minus response time for trials with a distractor that signaled low-reward) as a function of CHI-T score.

Correlation analyses showed a significant association between CHI-T scores and VMAC scores (*r* = .26, *p* < .001), with higher trait compulsivity being associated with greater attentional capture by reward-related stimuli. This result remained significant after controlling for the influence of psychological distress and impulsivity (*r* = .20, *p* = .008). Figure 4 shows the scatterplot of VMAC score as a function of CHI-T score.

## Discussion

The current study examined the extent to which a transdiagnostic measure of compulsivity, the CHI-T scale, was related to severity of symptoms across compulsive behaviors as well as compulsivity-related familial risk and reward-related attentional capture. This study also served to further explore psychometric properties of the CHI-T, this time applied to an online research study. The key finding was that total scores on the scale were significantly associated with severity of symptoms across a range of compulsive behaviors, including gambling, internet use, alcohol use, binge eating, and OCD-related compulsions. Furthermore, CHI-T scores were associated with familial risk of addictions as well as familial risk of OCD and related conditions, but not familial risk for psychosis/schizophrenia. These relationships were demonstrated using conventional correlations but also controlling for inter-dependence of variables using partial least squares (PLS). Finally, higher CHI-T scores were associated with greater reward-related attentional capture, implicating this as a core cognitive process that may contribute to a range of compulsive tendencies.

The finding that CHI-T score correlated with symptom severity across different compulsive behaviors adds to a previous study of CHI-T by extending its convergent validity with a wider range of behaviors, particularly Internet use and eating, and showing that it can also be used in online studies (whereas the initial validation study was in-person).^5^ As predicted, CHI-T was associated with familial risk of addiction and OC related disorders, highlighting the potential use of this scale as a measure that is sensitive to individual variations in compulsivity-related risk. For instance, CHI-T may be useful in examining how different risk factors interact to increase risk of developing a range of compulsive disorders in the general population. This avoids confounds common to studies that use clinical samples (eg, medication, chronicity, etc.). Also, this avoids the problem of having to use different scales for each behavior, which could be differentially sensitive to gauging variations in risk (especially at lower end of the continuum).

Finally, the finding that higher levels of trait compulsivity on the CHI-T were related to greater reward-related attentional capture implicates attentional sign tracking as a cognitive process that may be involved in a range of compulsive symptom types. This finding will allow human compulsivity research to draw upon the wealth of knowledge that has been derived from animal studies on sign-tracking, including the associative processes that underlie it, the neurological underpinnings, factors associated with risk (eg, early trauma, adolescent cannabis use, impulsivity, genetics), and potential targets for behavioral and pharmacological interventions.^15^ ^,^^16^ ^,^^43^ ^–^^46^

Several limitations should be considered regarding this study. The survey was conducted online, with all the inherent limitations thereof. For example, online assessment is unlikely to be as accurate as in-person clinical assessment in terms of precise quantification of psychiatric symptoms. Nonetheless, the study demonstrates the feasibility of using the current scale for online research. The survey respondents may have had participation bias, including due to the nature of the study advertisements, and thus the results may not generalize to the background population or other cohorts. Another limitation related to the online method is the relatively high number of participants (around 15%) who did not perform above chance level 50%. The high error rate may be related to the online nature of the study, in which participants are not supervised and thereby may be less attentive than in a strictly controlled lab setting, especially as the cognitive task was administered at the end of a 40-min questionnaire battery. Nonetheless, given that the learning that drives the VMAC effect should draw attention away from the target, then the relatively high error rate is not unexpected, especially in light of the variant used here, in which punishment of incorrect responses did not occur. Future studies using this task may benefit from exploring how errors themselves are related to compulsivity. Another limitation of the study is that we did not obtain more detailed background demographic information about the participants, such as levels of education. Finally, future research will benefit from comparing reward-related attentional capture with other cognitive measures that have commonly been found to be associated with compulsive symptoms in OCD, such as attentional set-shifting deficits and avoidance, habit, and/or reversal learning abnormalities.^47^ ^–^^50^

In summary, this study demonstrated that a transdiagnostic compulsivity scale was sensitive to a range of compulsive symptom types, and to family history of compulsive symptoms, controlling for general distress. Transdiagnostic compulsivity was also significantly related to reward-related attentional capture, a cognitive process that may thus play a key role across different compulsive disorders. Because transdiagnostic compulsivity is a relatively neglected research topic, we call for more research in this area, which might also explore biological underpinnings and genetics.

## Footnotes

1 For the internet use area, participants answered only 3 questions, on time spent, anxiety if prevented, and strongest urge (items 1, 5, and 6 of the BATCAP scale).

2 In Le Pelley et al.’s original version of the task, incorrect responses resulted in loss of the amount that would otherwise have been won as a reward. By contrast, in the current version of this task errors did *not* result in losses. This modification was made to ensure performance was less likely to be confounded by loss-related sensitivity and/or processes, as these are not central to sign-tracking.

## Supplementary material

For supplementary material accompanying this paper visit https://doi.org/10.1017/S1092852919001330. click here to view supplementary material
