# Supplementary material for: Compulsivity is measurable across distinct psychiatric symptom domains and is associated with familial risk and reward-related attentional capture
Source: CNS Spectr. 2019 Oct 24;25(4):519–26. doi: 10.1017/S1092852919001330 (PMC7115959; doi:10.1017/S1092852919001330)
Supplement: Supplementary file 1 [file S1092852919001330sup001.docx]

**Supplementary materials**

**Family history of compulsive behaviors scale**

Please indicate whether, as far as you know, you have a positive family history (among first degree biological relatives) of any of the behaviours/conditions listed below:

|  | Yes – one relative | Yes – multiple relatives | No | I don’t know |
| --- | --- | --- | --- | --- |
| Nicotine dependence |  |  |  |  |
| Alcohol use problems |  |  |  |  |
| Cannabis use problems |  |  |  |  |
| Other substance use problems |  |  |  |  |
| Gambling |  |  |  |  |
| Binge eating |  |  |  |  |
| OCD (contamination/washing) |  |  |  |  |
| OCD (ordering, arranging, checking, or counting) |  |  |  |  |
| OCD other (other OCD-related disorder or behaviour) |  |  |  |  |
| Hoarding disorder |  |  |  |  |
| Obsessive-compulsive personality disorder |  |  |  |  |
| Tourette’s or tic disorder |  |  |  |  |
| Body-focused repetitive habits (e.g., nail-biting, skin-picking, hair-pulling/trichotillomania) |  |  |  |  |
